# Field-based methods for measuring greenhouse gases emissions from on-site sanitation systems: A systematic review of published literature

**DOI:** 10.1016/j.heliyon.2023.e19947

**Published:** 2023-09-07

**Authors:** Prativa Poudel, Anish Ghimire, Guy Howard, Barbara Evans, Miller A. Camargo-Valero, Freya Mills, Olivia Reddy, Subodh Sharma, Sarana Tuladhar, Abraham Geremew, Kenan Okurut, Baba Ngom, Manish Baidya, Sheila Dangol

**Affiliations:** a Department of Environment Science and Engineering, Kathmandu University, Nepal; b Aquatic Ecology Centre, School of Science, Kathmandu University, Nepal; c Department of Civil Engineering and Cabot Institute for the Environment, University of Bristol, Bristol BS8 1TR, UK; d WASH Research Group, School of Civil Engineering, University of Leeds, Leeds LS2 9JT, UK; e BioResource Systems Research Group, School of Civil Engineering, University of Leeds, Leeds LS2 9JT, UK; f Departamento de Ingeniería Química, Universidad Nacional de Colombia, Campus La Nubia, Manizales, Colombia; g Institute for Sustainable Futures, University of Technology Sydney, 235 Jones St, Ultimo, NSW, 2007, Australia; h College of Health and Medical Sciences, Haramaya University, Dire Dawa, Ethiopia; i Department of Civil and Environmental Engineering, Kyambogo University, Kampala, Uganda; j Laboratoire Sciences et Techniques de l’Eau et de l’Environnement (LASTEE), Ecole Polytechnique de Thies (EPT), Thies, Senegal

**Keywords:** Greenhouse gas emissions, Onsite sanitation, Pit latrines, Septic tanks

## Abstract

On-site sanitation systems (OSS) are a source of greenhouse gas (GHG) emissions. Although efforts have been made recently to measure and quantify emissions from septic tanks using various field-based methods, the vast majority of published literature reporting GHG emissions from OSS units (e.g., pits and tanks) is based on non-empirical evidence. This systematic review presents an overview and limitations of field-based methods used for the quantification of GHG emissions from OSS**.** Papers published in English were searched in three databases: Google Scholar, PubMed, and Directory of Articles and Journals. Peer-reviewed papers that reported field-based methods applied to containment units in OSS were included in this study. Only eight out of 2085 papers met the inclusion criteria with septic tanks as the sole technology reported and were thus, considered for the review. Most of the studies have been conducted in middle- and high-income countries. Field-based measurements of GHGs are conducted using a flux chamber (FC) and the most commonly used FC methods are (a) the modified simple static FC, (b) automated static FC, and (c) floating FC. Data reported in published studies do not provide sufficient information on the calibration and validation of the results from the FCs used. The complex FC designs, laborious fieldwork operations, and reliance on expensive, specialist equipment, suggest that such methods may not be suitable in Low and Middle-Income countries (LMICs), where resources and access to laboratory facilities are limited. Also, the complexity of pits and tank typology in LMICs (i.e., unstandardised designs and sizes) may be a challenge to the use of FCs with fixed dimensions and set operational conditions. The variation in the quantification methods and resulting emission rates among the studies indicates that gaps prevail in the use of existing methods. Therefore, there is still a need for a simple field-based, easily adaptable FC method with adequate calibration and validation that can help in reliably quantifying the emissions from different OSS in any LMICs.

## Graphical abstract

**Figure:**
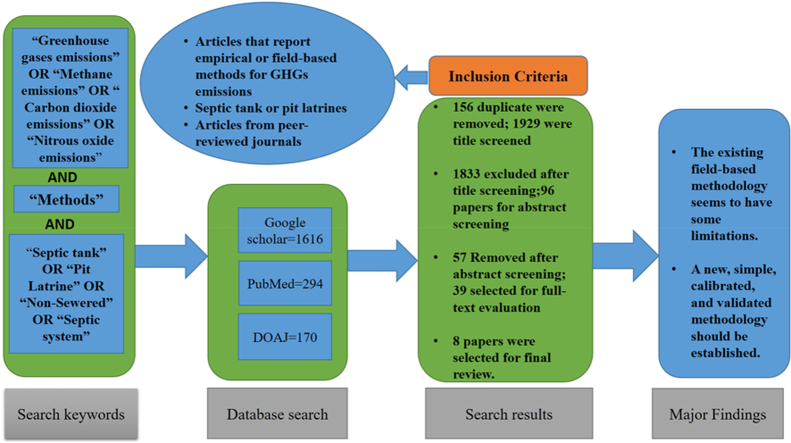

## 1 Introduction

Human waste management has always been crucial to protect the environment and human health [1]. Onsite sanitation systems (OSS) have played vital roles in this crucial issue [2]. The United Nations Sustainable Development Goals (SDGs) have raised the priority given to end open defecation (SDG6 – clean water and sanitation), with OSS at the forefront in LMICs [3]. With 3.3 billion users, the spread of OSS is substantially larger than sewer-based sanitation [4]. Typically, sustainable and well-managed OSS include the collection, storage, transport, treatment and safe disposal or reuse of human excreta and wastewater at or near the source where it is generated [4]. For excreta collection, this may include the use of pits (single, dual, pour flush, ventilated improved, etc., pit latrines), tanks (holding, septic tanks etc.) or containers (composting toilets., container-based sanitation).

**Fig. 1:**
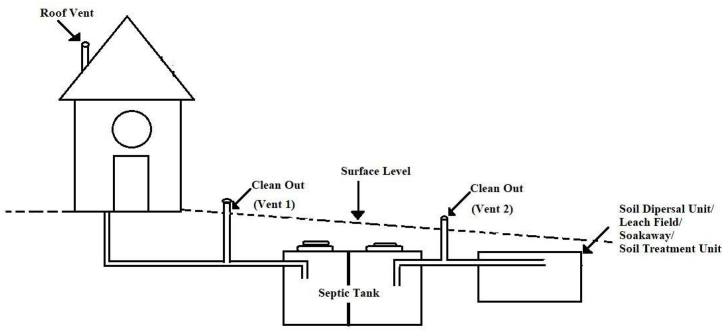
Different components of the septic system.

The increasing dependence on OSS technologies, and the lack of local expertise for the correct design, construction, operation and maintenance, may have some negative consequences, such as increased microbial groundwater and soil contamination [[5], [6], [7], [8], [9], [10]]. In addition, pits and septic tanks (ST) have been reported as sources of non-negligible GHGs emissions [[11], [12], [13]]. A recent meta-analysis indicated that the global methane emission from OSS could be as high as 4.7% of global anthropogenic methane emissions in 2020 [14]. Reid et al. [12] predicted that GHGs from pit latrines account for one percent of global emissions. More use of OSS to ensure that SDG6 is met may lead to an increase in GHG emissions [15]. In a typical onsite sanitation unit, anaerobic processes are dominant in the sludge layer and water column inside the holding and septic tanks and wet pits; whilst aerobic processes may prevail in soakaways, leach fields and dry pit latrines [16,17]. Though the dominant process occurring inside any type of containment is debatable, we can argue that a combination of anaerobic and aerobic digestion processes is responsible for the nature and extent of GHG emissions. It has been reported that septic tanks and pit latrines mainly emit Methane (CH_4_), and Carbon dioxide (CO_2_) [16,17], whilst emissions of nitrous oxide (N_2_O) often occur in soil treatment units (STUs) and pit latrines [[18], [19], [20]]. A septic system comprises a ST followed by a STU (Fig. 1). According to the Joint Monitoring Program (JMP), “Septic tanks are the containments to contain and treat excreta in situ and should have at least two chambers separated by a baffle and a T-shaped outlet pipe to reduce the scum and solids that are discharged. The effluent should infiltrate into the subsurface through STUs like soak pit or leach field or soil dispersal unit, or discharge to a sewer system” (Fig. 1) [21].

**Adapted from:** Diaz-Valbuena et al. [18] and Truhlar et al. [20].

The quantification of GHGs emissions for national inventories is based on modelling and relies on secondary data regarding per capita contributions of organic matter reported as Biochemical Oxygen Demand (BOD) and an Emission Factor (EF) that represents the conversion of organic matter into GHGs. These quantification models reported in most of the earlier studies are based on the methodology set out by the *Intergovernmental* *Panel on Climate Change* (I*PCC)* and the United States Environmental Protection Agency (USEPA) [11,22]. The IPCC methodology uses major three tiers of method – i.e., Tier I, Tier II and, Tier III. Tier II and III are considered as more accurate methods and are used by the nations that have their own data set. While the Tier I methodological approach is used by nations where the national level data set is unavailable. In such countries, field-level data should be generated to help them report more accurate emissions [11]. However, studies that have used field-based measurements are very small in number and have been undertaken in diverse settings using different techniques [23].

Improving estimates of GHG emissions from OSS using first-hand data is important to better quantify its contribution to overall emissions. This systematic review aims to give an overview and critically assess the reported field-based methodologies for quantifying GHGs from OSS and to identify what further methodological developments should be prioritised.

## 2 Materials and methods

### 2.1 Search strategy

We followed the Preferred Reporting Items for Systematic Reviews (PRISMA) guideline [24], using three online databases: Google Scholar, PubMed, and the Directory of Articles and Journals (DOAJ). Ms Poudel and Ms Tuladhar conducted the searches in 2022, including works published in October 2022. The keywords, greenhouse gas emissions, methane emissions, carbon dioxide emissions, nitrous oxide emissions, septic tanks, pit latrines, non-sewered, and septic systems were used to search published work (Table 1). Then, the identified keywords were cross-checked across all three selected electronic databases. The search outcomes were exported into Microsoft Excel®, duplicate titles were screened and removed. Additionally, all identified articles' reference lists were searched for additional articles. The titles of all the articles were screened, and those appearing relevant were further examined by reading the abstracts. The articles having pertinent abstracts were read to finalize the initial search.

**Table 1:** Search strings for databases.

Searched database	Exact search string
Google Scholar, PubMed, DOAJ	"Greenhouse gases emissions" OR "Methane emissions" OR "Carbon dioxide emissions" OR ″ Nitrous oxide emissions" AND "Method" AND "Septic tank" OR "Pit Latrine" OR "Non-sewered" OR "Septic system"

### 2.2 Inclusion and exclusion criteria

Major inclusion criteria were: a) articles that report field-based methods, and b) articles that report field methodology for emissions measurement from a septic tank or pit latrines or any other onsite sanitation technologies that serve at the household level. All published articles from peer-reviewed journals and written in the English language were included. Articles that include methodologies using theoretical models, letters from editors, case reports, conference abstracts, and unpublished studies were excluded.

### 2.3 Outcome measures

The major outcome measures included the method used for the sampling of gas, the method and tools used for gas sample analysis, and limitations in the current methodologies.

## 3 Results

### 3.1 Search results

**Fig. 2:**
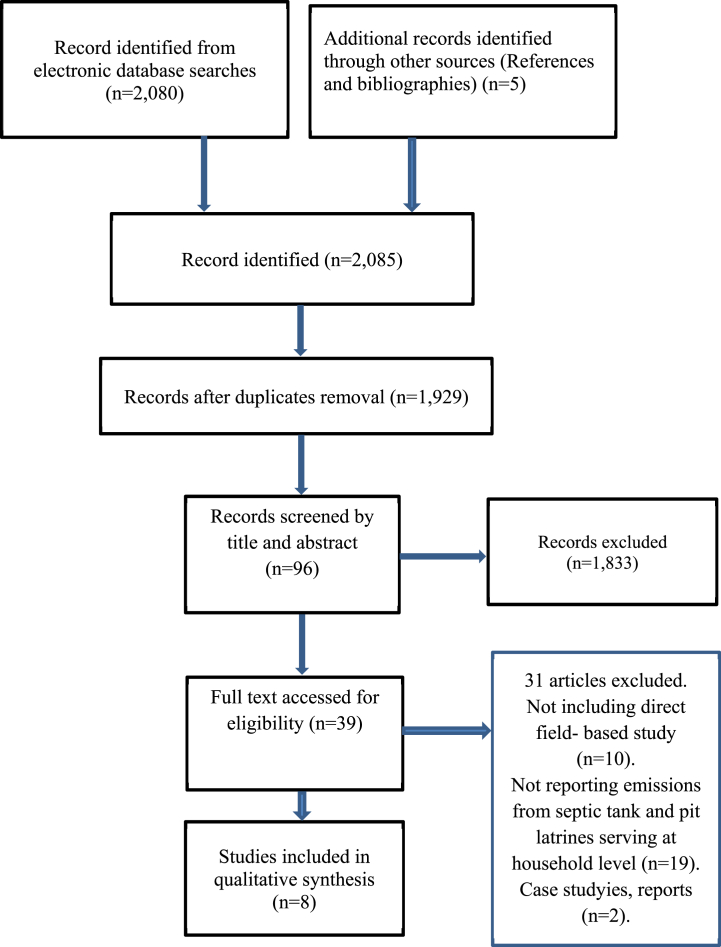
PRISMA flow diagram for the search of studies on field-based GHG quantification methods.

A total of 2085 studies were identified in the selected electronic database (Google Scholar = 1616; PubMed = 294; DOAJ = 170; and other sources – i.e., bibliographies and references = 5) (Fig. 2). By removing duplicated hits, we excluded 156 papers. Of the 1929 non-duplicated papers, 1833 were excluded at title screening. A total of 39 papers were screened as relevant from 96 papers after reading the abstracts. Finally, after the full-text screening, eight papers were found to meet the eligibility criteria; therefore, they were included in the critical review of field-based quantification methodologies for GHGs from on-site sanitation units.

### 3.2 Study characteristics

The papers finalized for this review were the only articles that reported GHGs from OSS. Out of eight papers, four included fieldwork from the United States of America (USA), one from Vietnam, and three from Ireland. All eight papers reported the emissions from a single containment type – i.e., septic tank (Table 2).

**Table 2:** Information extracted for the systematic review.

Reference	Location	Number of sites	Containment type	Gas measured from	Sampling device/method/tool
Winneberger [28]	USA	7 septic tanks	Septic tank	Containment	Submerged inverted steel bowls
Diaz -Valbuena et al. [18]	California, USA	8 septic tanks, 2 vents system and 2 soil dispersal systems; a total of 8 septic systems	Septic tank	Containment, vent or clean out port of the septic system of soil dispersal system	Modified flux chamber (FC): modified soil-plant-based system, connected to a fan that is supported by the power backup
Truhlar et al. [30]	New York, USA	9 leach fields, 9 vents, and 9 sand filters	Septic tank	Vents, leach field, and sand filter	The gas flux chamber constructed following the methods of Molodovskaya et al. [31]
Somlai-Haase et al. [26]	Westmeath, Ireland	1 septic tank connected to soak-away	Septic tank	Soak away and control only	Multi-chamber automated soil flux chamber
Somlai-Haase et al. [27]	Westmeath, Ireland	1 septic tank connected to soak-away	Septic tank	Soak away	Multi-chamber automated soil flux chamber
Truhlar et al. [20]	New York, USA	3 septic systems		Leach field	The gas flux chamber constructed following the methods of Molodovskaya et al. [31]
Huynh et al. [29]	Hanoi, Vietnam	10 septic tank	Septic tank	Containment	Floating flux chamber
Knappe et al. [25]	Limerick, Ireland	2 decentralised domestic wastewater treatment plants	Septic tank	Containment, vent port, soil dispersal system	Integrated and automated soil gas flux measurement systems, and hot wire anemometer for the emissions from the vents

One out of eight studies measured GHG emissions from the septic tank (ST) and soil treatment unit (STU) [25]. Three studies have measured GHG emissions from STU [[25], [26], [27]]. Two studies have evaluated the emission from STs [28,29]. The remaining two articles estimated GHG emissions from ST, STU, and the vent pipe of the septic system [18,30].

### 3.3 Gas collection techniques and uses in septic systems

Direct field-based GHG estimates for OSS like septic systems and pit latrines are rarely reported in the literature. Gas flux measurements are reported in various environmental systems like wetlands, lagoons, and ponds, among others [23] and in off-site faecal sludge treatment systems based on composting, wastewater stabilisation ponds and land disposal [34]. The methodology that has been used in the different interfaces – i.e., the water-, soil-, and waste-air interface for capturing gaseous emissions, which were previously used for other systems, have been adapted for measuring the GHG emissions from OSS, with the FC method being the most commonly used.

Winneberger in 1984 was the first to introduce the use of a FC method in septic tanks in the form of submerged inverted bowls FC [28]. There were no studies conducting direct measurements until Diaz-Valbuena et al. [18]; this study adapted FCs previously used for the soil-plant interface [18]. A similar FC is used by Truhlar et al. [20,30]. FCs used were modified static FCs [20,30].

Indeed, the FC methods were found to be the most prominently field-based method used. FCs can also be an automated static cylindrical chamber used to determine the emissions from the ST and STU [20,[25], [26], [27]]. Most of the studies have preferred static chambers. Similarly, floating FCs are also found to be adopted. Static chambers and floating chambers are similar in construction but the major difference is that the former is kept at a fixed position throughout the sampling period while the latter is allowed to float to collect gases [18,30]. The design and construction of the FC for each component of a septic system like ST, STU, and vent or any clean-out port of the septic system are different. Typical FCs are only used for ST and STU. For the vent and the clean-out port, different modified and fitted equipment are connected to anemometers and thermometers to also assess gas flow rates. The body of the typical flux chamber, used for ST or STU, is constructed from Polyvinyl Chloride (PVC) with a cylindrical shape – i.e., diameter of 0.15–0.3 m [18,20,25,29]. The cap used to cover the chamber is also made from PVC and fitted with 3/18 inches brass Swagelok® connectors for the connection of the sampling tube with rubber septum [18,20,30]. The cap is also vented with the thermometer and the vent for maintaining the pressure equilibrium. Such pressure vents are either made from vinyl tubing or aluminium connected to plastic connections [18,30].

The use of a static chamber for measuring GHG emissions from ST and STU has been reported in five studies. The insert was permanently fixed to 5 cm on the soil to stabilize the FC for STU. Inserts used in the tanks were 10 inches in diameter with PVC legs to support the insert [18,25,30]. Unlike static chambers, only one study has shown the use of floating chambers connected to the sample tube and pressure equilibrium pipes. The connection of the fan, supplied with the 12V battery, was observed in four studies. The main aim was reported to ensure the mixing of the gases [18,29].

### 3.4 Gas sample collection methods

Five out of eight studies used manual sampling methods for quantifying the composition of GHGs collected. Syringes are used to collect the gas samples and transferred to pre-evacuated vials through a syringe from a septum that is installed for sample collection on the FC [18]. The capacity of the syringe and the time intervals for consecutive sampling vary in different studies. 12 mL, 20 mL, 24 mL, 500 mL, and 1 L syringes are the most common syringes used for gas sampling [18,20,29,30]. The sampling interval time between each sampling ranged from 2 min to 10 min for 30 min long [18,29,30]. Generally, such frequency and total duration of sampling were found to be determined based on the chamber dimensions [32]. A study by Winneberger in 1984 did not mention the sampling method [28]. The samples were stored either in pre-evacuated vials, a Teflon bag or a large foil sampling bag to safely transport the gas samples to the laboratory for further analysis. In three among eight studies that used the automated chambers, a similar system was found to be deployed for hourly measurement and a 3-min measurement period using portable gas samplers [[25], [26], [27]].

### 3.5 Gas sample analysis

Four studies have reported the analysis of collected gas using gas chromatography (GC). A gas chromatograph coupled with an electron capture detector (ECD), flame ionization detector (FID), or thermal conductivity detector (TCD) was found to be used to analyse the sampled composition of GHGs (CH_4_, CO_2_, and N_2_O) [18,20,25,30]. FID detector was used for detecting methane (CH_4_) and carbon dioxide (CO_2_), and ECD for N_2_O [30]. TCD detector was used by Diaz-Valbuena in 2011 for N_2_O detection [18]. In the study conducted by Winneberger, gas sample analysis was conducted via spectrophotometric and iodometry tests [28]. Portable devices for gas analyses are also reported in a few articles; devices like the Portable Photosynthesis System attached to a LI6250 CO_2_ Analyser (LI-COR, Lincoln, NE) were used by Truhlar in 2016 and 2019 [20,30].

Similarly, an automated cylindrical chamber system (LI-8100A Automated Soil Gas Flux System, LI-COR Biosciences, Inc.) consisting of a non-dispersive infrared gas analyser, a multiplexer, and two automated opaque long-term chambers (LI8100-104, LI-COR Biosciences, Inc.) were used to analyse the long term soil flux of CO_2_ [26,27]. For methane, an ultraportable gas analyser (UGGA 915-0011, Los Gatos Research) was integrated into the gas loop [27]. Table 3 summarises the various analysis methods used in the previous studies.

**Table 3:** Methods used for the analysis of collected gas samples.

Author(s)	Gas sample analysis methods	Gases
Winneberger [28]	Mass spectrometry and iodometry	CH_4_, H_2_S
Diaz-Valbuena et al. [18]	Shimadzu gas chromatograph (Model GC-2014) with a 63Ni ECD, FID, and TCD linked to a Shimadzu autosampler (Model AOC-5000)	CH_4_, CO_2_, and N_2_O
Truhlar et al. [30] Truhlar et al. [20]	Gas chromatograph (Model 6890 N GC/ECD, Agilent Technologies Inc., Santa Clara, CA) using an FID and an ECD	CH_4_, N_2_O
Truhlar et al. [30] Truhlar et al. [20]	Portable Photosynthesis System attached to a LI6250 CO_2_ analyzer (LI-COR, Lincoln, NE)	CO_2_
Somlai-Haase et al. [26] Somlai-Haase et al. [27] Knappe et al. [25]	Automated cylindrical chamber system (LI-8100A Automated Soil Gas Flux System, LI-COR Biosciences, Inc.) consisting of a non-dispersive infrared gas analyzer, a multiplexer, and two automated opaque long-term chambers (LI8100-104, LI-COR Biosciences, Inc.)	CO_2_
Somlai-Haase et al. [27]	Ultraportable Greenhouse Gas Analyser, model 915-0011	CH_4_
Knappe et al. [25]	CH_4_ (UGGA 915-0011, Los Gatos Research) gas chromatograph (Clarus 500, Perkin Elmer) equipped with capillary columns (Elite-Plot Q), FID for CH_4_, and ECD for N_2_O	CH_4_, N_2_O
Huynh et al. [29]	N2O in the collected samples were determined using a gas chromatograph (Shimadzu GC-2014) with a flame ionization detector, thermal conductivity detector, and electron capture detector, respectively.	CH_4_, CO_2,_ N_2_O, CO, NH_3_
Huynh et al. [29]	Portable gas analyser (PG300, HORIBA)	CH_4,_ CO_2_

### 3.6 Data analysis for GHG emission flux calculations

Depending on the section of the septic system where the concentration was measured, three approaches have been reported in the literature. Four studies used the mass balance approach for the calculation of GHG emission data [20,[25], [26], [27]]; three used the linear plot method for the emission rate calculation [18,29,30]; and one study used data generated from spectrophotometric and iodometry tests [28].

As a part of the linear plot approach, the concentration of the gases was converted into mg/m^3^ concentrations by using Equation (1) [18,29,30]:.

(1) $$Gas\;concentration\left(\text{mg}/m^{3}\right)=\frac{\left(Cppm/10^{6}\right)\;\left(MW\right)\left(1000mg/g\right)}{\frac{RT}{P}}$$

*C*_*ppm*_ is the concentration of gas in ppm.

*MW* is the molecular weight of the gas under consideration (g/mol)

*R* is the gas constant (0.000082057 atm m^3^/mol·K)

*T* is the absolute temperature (K)

*P* is the absolute pressure of the gas (atm)

Gas concentration values (mg/m^3^) from each measurement event were then plotted as a function of time. The slope *m*, in units of mg/m^3^min derived from a linear fit of the data was then used to compute the flux using Equation (2):

(2) $$E=\frac{m*1440*10^{3}*V_{FC}*A_{Comp}}{A_{FC}}*N$$

where,

*E* is the emission rate per capita (g capita^−1^ day^−1^)

*m* is the emission rate based on the inner concentration of the floating chamber (mg/m^3^ -gas/min)

1440 is minutes in a day (min/day)

*V*_*FC*_ is the chamber headspace volume (m^3^)

*A*_*cont*_ is the surface area of the containment unit (m^2^)

*A*_*FC*_ is the area covered by the floating chamber (m^2^)

If the regression coefficient (*R*^*2*^) value was less than 0.75 then the measurements were discarded in a report by (Truhlar et al., 2019).Unlike the first approach, the second approach has been reported to be more appropriate to conduct in vents or clean-out ports [18,25,30]. Clean-out port or roof vents are part of the building codes that allow the gases formed inside the septic tanks and the dispersal unit to evacuate. It is assumed that for a completely sealed septic system all the gases escape via the vents or clean-out ports. The emission rate ($E_{T}$) from the septic tank or pit latrine is calculated by multiplying the surface emission from gas ($E_{s}$) and the total surface area of the faecal sludge or septage holding tanks (i.e., septic tanks or pits) given in Equation (3):

(3) $$E_{T}=E_{s}*A$$

where,

$E_{s}$ = Emission rate from the onsite containment like septic tanks and pit latrines (mg of GHG/m^2^/d)

A = Surface area of a septic tank or pit latrine (m^2^)

Surface emissions from gas are calculated as Equation (4):

(4) $$E_{s}=\frac{Q_{gas}*C}{B}$$

where,

$Q_{gas}$ = Volumetric Gas flow rate (m^3^/s or L/s)

C = Concentration of gas in the gas mix (mg/L)

B = Cross-sectional area of the gas flux chamber.

Volumetric gas flow rate is calculated as equation (5):

(5) $$Q_{gas}=V*A_{v}$$

V = Flow velocity of the gas in the vent (m/s)

$A_{v}$ = cross-section area of the vent pipe (m^2^**)**

Although the type of FC used in past research was different, the main principle of the FC is observed to remain constant.

The mass balance approach is another method of calculating the emissions rate from the ST and the STUs.

Fluxes F [μmolm^−2^s^−1^] via a mass balance approach is represented by Equation (6).

(6) $$F=\frac{V_{cham}*P^{0}\left(1-x_{w}\right)}{RsT^{0}}*\frac{\partial X_{c}\left(t\right)}{\partial t}$$

where,

$V_{cham}$ = volume of the chamber *V*_*cham*_ in m^3^

$P^{0}$ = atmospheric pressure at the beginning of the measurement ins Pascal

$x_{w}$ = air water vapor mole fraction in mol mol−1

R = the universal gas constant R-value

R = soil collar surface area

$T^{0}$ = the absolute temperature at the beginning of the measurement in kelvin

∂Xc/∂t = the initial change of chamber water vapor corrected CO_2_ mole fraction ∂Xc/∂t [μ mol mol−1 s−1]

$X_{c}\left(t\right)$ is observed to be calculated using an empirical exponential regression model which is fit to measured gas concentration data as in Equation (7):

(7) $$x_{c}\left(t\right)=x_{o}-x_{t}∙e^{\left(-a\left(t-t_{o}\right)\right)}+x_{x}$$

where Xc(t) is the instantaneous water-corrected chamber CO_2_ mole fraction.

*X*_*0*_ is the value of *X(t)* when the chamber closed,

*X*_*x*_ is a parameter that defines the asymptote, μmol/mol

*a* is a parameter that defines the curvature of the fit (s^−1^)

Now, TheEmission rate was converted to per capita,ass equation rates *E*[g capita^−1^ day^−1^] using Equation 8

(8) $$E=\left(A_{soak}M*F\right)/N$$

E = Emission rate of the gases

M = Molar mass of gas

N = Number of occupants

F = Flux in μmolm^−2^s^−1^

### 3.7 Limitations of the field –based methods

The FC method is found the most prominent field-based method, for measuring GHG emissions from STs. However, this method was critically assessed to determine the limitations. Limitations to the method including from collection of the gas and analysis of the samples were observed. Comparison was made between the calculated mass emission rate of gases reported in previous studies. The consistency in the emission rate was lacking in similar kinds of containments. One of the probable causes of this inconsistency in the emission rate obtained may be due to variations in the design and operation of the FC in each study. For instance, Winneberger in 1984 performed an experiment that consisted of a simple inverted bowl that allowed the CO_2_ to dissolve back to the liquid surface which undervalued the emissions made from the tank (i.e., 5–6 g CO_2_ capita^−1^ day^−1^) [28]. Similarly, Huynh et al. [29] inserted 5 mm of the bottom of the FC into the septage which may not have been enough to avoid the dispersion of the gases. For these reasons and drawbacks, obtained emissions may have been underestimated. Limited insertion of FC to the septage or longer contact with the surface liquid might have reduced the concentration of the gases inside FC through leakage or dissolving back to the septage. Along with this, there might be several other factors that limit the results. The detection of the leakage either in the cover/cap of the FC or the connectors is not mentioned in any of the studies. Most of the flux chambers were fitted with a fan with a power supply [18,29,30]. The fitting of the fan must have led to multiple holes in the flux chambers that increase the chances of leakage. Such fans not only can cause leakages but the requirement of heavy power backup may cause less feasibility in geographically challenging areas, this can reduce the adaptability of the FC in all geographical areas. Diaz-Valbuena et al. [18] used some PVC legs to support the inserts and the FC. These legs were designed for fixed-depth septic tanks. Containments in LMICs are diverse in size and design. This would again lead to lower adaptability in LMICs.

Calibration of the FC can play a significant role in the determination of precise emission rates. Calibration of the FC is essential specifically when the chamber is adapted from, some other field. The studies might well have calibrated their FC but none of the papers covered in this review have mentioned that. Since most of the FCs were adapted from the soil-plant base interface, it was necessary to calibrate it in the liquid–air context. Calibration in terms of flow rate and concentration is not mentioned in any of the papers included. Automatic flux chamber system used in Knappe et al. [25] studies are designed and constructed for the solid-gas phase, which was adapted without any prior verification and calibration. Besides the limitation of the sampling using FC, what current studies lack is the representativeness of the data set. Most of the studies done are one-time sampling rather than the seasonal sampling. Only Knappe et al. have done seasonal sampling. The seasonality affects the hydrology, groundwater changes and ambient temperature. Inundation of the containment like pit latrines and holding tanks, making the sludge moist may increase the moisture inside the containment [12,33]. The moister sludge the higher GHG emissions than the drier sludge that are evident in treatment systems stabilising faecal sludge from ECOSAN latrines [34]. Therefore, studies that capture seasonal variation are important to better characterise and understand GHG emissions.

The resource cost of the FC method from the sampling to sample analysis was also assessed qualitatively. Sample analysis methods majorly involve the use of Gas Chromatography (GC), which is based on expensive analytical instruments with specialist detectors like ED and FID. Such analytical methods, expensive and inaccessible to many, may be difficult to adapt in resource-limited settings in LMICs.

## 4 Discussion

Our systematic review indicates there is limited published evidence on field-based methodologies for the measurement of GHGs from OSS. Unlike direct field-based measurement, a widely used and accepted theoretical modelling approach is provided in the IPCC GHG inventory guidelines 2006 with an amendment in 2019. The stoichiometric relationships between the organic loading in terms of BOD provided by IPCC have been adopted globally for quantifying the national emission inventories from various sectors, which also include wastewater treatment plants (WWTP) and discharge of wastewater as a key component [11,35]. IPCC methods only cover the emission of CH_4_ and N_2_O and provide the benchmark for the CH_4_ and N_2_O emissions from septic systems. CO_2_ emission from wastewater is considered biogenic as per the IPCC guidelines and is not incorporated in the inventory. IPCC is a modelling approach, and the validation of the modelled methodology can only be done by field-based measurements. The comparison made between the emission rate generated from the IPCC method and field-based measurement shows that calculated emission rates are overestimated compared to field-based measurement [18,29,30]. However, the IPCC method provides a benchmark of the emissions from the containments. Also, itt may be possible that the field-based method is not sufficiently designed and implemented to provide more robust and comparable data. Many limitations, in terms of study design, design construction and operation of FC, cost, sample analysis etc. are observed in flux chambers that have reduced the adaptability in resource constraints and geographically challenging areas in some countries.

In terms of study design, the number of samples taken in the available studies is limited. The maximum number of STs included as a sampling site is ten. These sample sizes may be justifiable, if the character of all the samples are similar. For instance, all septic tanks included in the study by Diaz et al. [18] and Huynh et al. [29] are well-engineered septic tanks. However, these numbers might not be applicable if the containment character is different from one household to another. Holding tanks and pit latrines in LMICs are not engineered containments that vary in their design and operational conditions [29]. This number of samples in such a context must be high enough to conclude.

The design and modification needed for FCs would depend on the actual nature of the emission source which may be a solid–gas phase or liquid-gas phase [36]. Since the specific calibration procedure may vary depending on the application and design of the flux chamber being used. The calibration of FC is important to validate its applicability in field conditions [37]. The FC method used to measured GHG emissions in onsite sanitation units is adapted from the solid-gas phase that is used in soils and the closest approximation would be for use in STUs. These adapted FCs lack calibration and validation before measuring the gases. The FC is adapted from other fields – i.e. solid-gas phase and must be calibrated when used for the liquid-gas phase. Knappe et al. [25] and Somlai-hasse et al. [27] have used an automated soil-gas CO_2_ flux system, which is originally designed to measure emissions from the soil. But these studies have used the same system to quantify the GHG emissions from the septic tank (liquid-gas phase). But both the studies have failed to mention on the calibration while using in the septic tanks [[25], [26], [27]].

The use of a mehanical fan to homogenise the gas mix in the chanber's headspace requires an energy supply. This need for a power backup sets the limitation for geographical aspects where access to power supply is a challenge. Adding to this, moisture content inside the containment may damage electrical connections. The use of battery powered mechanical fans can be an alternative that shoulb be balanced against additional capital and operation and maintenance costs. Flux chambers that are designed for use in fixed-depth septic tanks may not be suitable in settings where tanks are not of a uniform size or do not conform to standard septic tank designs [18]. Some modification in terms of flexible inserts must be designed for the containment that is of varying depth. The methodology that exists now may not be applicable or adapted to various onsite sanitation types because of substantial differences in design, construction and operation.

The sampling cost remains a major factor in LMICs when it comes to replicating the methods reported in the literature. Automated flux chambers are expensive as they use sensitive gas detectors and analyzers (LI8100-104, LI-COR Biosciences) to measure gas composition onsite and in real-time [26,27]. Alternatively, methods including the collection and transport of gas samples for instrumental analysis, rely on access to laboratory facilities with equipment (GC/MS) and trained personnel to run the corresponding analytical tests; therefore, transportation costs, access to lab facilities, and the long terms preservation may again increase the cost of using this method in LMICs.

Most of the past studies are done in countries with low coverage of pit latrines and are limited to septic systems. Reid et al. [12] used a spatially explicit approach analysis, hydrological control, and country-specific emission factors to estimate global CH_4_ emissions from pit latrines. The study concluded that CH_4_ is responsible for 1% of the CH_4_ out of total GHG emissions globally. A recent study predicted that GHG emissions from OSS or non-sewered connections account for 4.7% of global emissions [13]. This suggests that GHG emissions from OSS have more significant contributions than it was realized. Likewise, in LMICs OSS like pit latrines or holding tanks are the most adopted and low-cost technologies. Methodologies used to date are designed and adapted for septic tanks and may not be directly applicable to pit latrines and holding tanks. Therefore, an appropriate method that can apply to almost every onsite sanitation technology is required. The new method in this context must be simple, cheaper, and most importantly adaptable in any geographic location and containment type.

## 5 Conclusion

The FC method is the most preferred method for determining GHG emissions from septic tanks. However, no field-based studies have been conducted to determine the emissions from other OSSs like pit latrines and holding tanks. Limitations in terms of design and operations, cost, and sample analysis are major factors that may reduce the replicability and adaptability of published field-based works to measure GHG from OSS, particularly when considering the variations in the design and operation of tanks and pits in LMICs. Therefore, we highlight the importance of designing, constructing, adequately calibrating, and validating a method for the field-based measurement of GHGs in OSSs. This can help to better assess emission factors and actual emissions rates from such sanitation systems, particularly in LMICs where we need to capture the various types of onsite sanitation units, which are presumably a major source of GHG emissions.

## Authorship contribution statement

AG, GH and MACV: Conceptualization, Methodology, Review & Editing; PP and ST: Data curation, Investigations, Writing, Writing- Original Draft; OR, BN, SD, BN and AG: Writing- Review & Editing; GH, FM, SS, BE, MACV and MB: Supervision, Review & Editing

## Funding

This work was supported, in whole or in part, by the 10.13039/100000865Bill & Melinda Gates Foundation under grant No: INV-015713. Under the grant conditions of the Foundation, a Creative Commons Attribution 4.0 Generic License has already been assigned to the Author Accepted Manuscript version that might arise from this submission.

## Data availability

Data used are either included or referenced in the article.

## Additional information

No additional information is available for this paper.

## Declaration of competing interest

The authors declare that they have no known competing financial interests or personal relationships that could have appeared to influence the work reported in this paper.
